# Patient-matched instruments versus standard instrumentation in total knee arthroplasty: a prospective randomized study

**DOI:** 10.1007/s00508-015-0703-0

**Published:** 2015-03-03

**Authors:** Andrej Molicnik, Jakob Naranda, Drago Dolinar

**Affiliations:** 1 Orthopaedic Department, University Clinical Center Maribor, Ljubljanska 5, 2000 Maribor, Slovenia; 2 Ljubljanska 3, 2000 Maribor, Slovenia; 3 Departement of Orthopaedic Surgery, University Medical Centre Ljubljana, Zaloška cesta 7, 1000 Ljubljana, Slovenia

**Keywords:** Total knee arthroplasty (TKA), Patient-matched instruments (PMI), Coronal alignment, Intraoperative blood loss, Operation time

## Abstract

**Background:**

Optimal positioning of implants and restoration of neutral mechanical axis are two primary surgical goals in total knee arthroplasty (TKA). Despite modern instruments and improved surgical techniques, malalignment remains an important cause of early failure after TKA. The aim of this prospective randomized study was to compare the value of a new patient-matched instrument system (PMI) (Signature^TM^; Biomet, Inc, Warsaw, Indiana) to that of standard TKA surgical instrumentation (STD) in terms of coronal mechanical alignment, time of operation and blood loss.

**Patients and methods:**

A total of 38 patients waiting for primary TKA were enrolled and randomized into two groups (19 PMI and 19 STD). Magnetic resonance imaging was performed in all patients in the PMI group, and specific instruments for the femur and tibia were designed preoperatively. All patients were operated on using the standard medial parapatellar approach with no use of tourniquet. Mechanical axis, time for the operation, and blood loss were evaluated.

**Results:**

Patients in both groups had comparable age, body mass index, preoperative mechanical axis, Knee Society Score, and level of hemoglobin. Postoperative results showed that the PMI group fell significantly closer to neutral mechanical axis (STD: 2.7 ± 1.7, PMI: 1.7 ± 0.9; *P* = 0.013) with no outliers and a reduced time for the operation. There was no difference in the evaluation blood loss.

**Conclusions:**

The use of PMI can contribute in achieving better mechanical axis with reduction in outliers and decreased operation time. Due to small differences between PMI and standard instruments, additional research are needed to confirm these preliminary results, and to discover potential benefits and functional improvements in the long-term outcome.

## Introduction

Total knee arthroplasty (TKA) is an effective intervention for relieving pain and disability in people with advanced osteoarthritis of the knee. This surgical procedure has undergone several improvements during its routine performance over more than 40 years, and it is becoming increasingly important to achieve improved patient satisfaction by providing good knee function, increased quality of life, long-term survival of the prosthesis, and a good cost-benefit ratio [1, 2]. Survival rates from 80 to 98 % have been reported for modern TKA [3–6], and approximately 25 % of all failures of TKA are related to malpositioning or malalignment [7–9]. Biomechanical studies have demonstrated a change of load distribution with 3° of varus or valgus [10], and there have been several reports suggesting that postoperative mechanical alignment outside this range (3° varus/valgus regarding to mechanical neutral) may be associated with poor survival of the prosthesis and long-term risk of revision [11–14]. General acceptance of optimal alignment of the component position is within this 3-degree range in coronal and sagittal planes [11], although several papers have raised questions about this [15].

The conventional operating method with standard TKA instrumentation, which relies on manual instrumentation using intra- and extramedullary (femoral and tibial) jigs and extensive visual referencing of bony landmarks [16], recorded excessive deviation from a neutral mechanical axis with the incidence exceeding 25 % even in some major arthroplasty centers [17–21]. This can also lead to inferior functional outcomes [22]. The novelty in TKA surgery in recent years has become a computer-assisted insertion of the prosthesis (computer-assisted surgery), which provides optimal determination of the mechanical axis and thus better postoperative biomechanics of the knee joint [23–25], but has limitations involving increased surgical time (pin placing, landmark registration), surgical costs, pin loosening, and pin-related bone fracture. It also demands a substantial learning curve [26]. However, a revolutionary approach in TKA surgery is represented by patient-matched instrumentation (PMI) based on the creation of specific three-dimensional (3D) models of a patient’s distal femur and proximal tibia on the basis of magnetic resonance imaging (MRI) or computed tomography scan of a the patient’s hip, knee, and ankle. The proper software is used to create a virtual 3D reconstruction of the knee joint and to determine mechanical axis, implant sizing, plan bone cuts as well as positioning and alignment of the prosthesis. With the use of rapid prototyping technology, custom pin-positioning guides that work with standard instruments and implants are manufactured preoperatively to perfectly match the individual anatomy of the arthritic knee joint. Preliminary operative results strongly suggest the advantage of using PMI, which enables optimal resection and assures accurate neutral mechanical axis. Additionally, the operation has been reported to be faster and less invasive (no intramedullary violation, less time in hardware positioning); therefore, the risk of infection, blood loss, and perioperative complications (e.g., fat embolism) could also be minimized. However, there are only a few clinical studies supporting the efficiency of this novel approach [26, 27].

The aim of the current study was to compare the results of postoperative coronal alignment obtained by PMI (using Signature^TM^ system, Biomet, Inc., Warsaw, IN, USA) and that of standard TKA instrumentation and also to compare the operative time and blood loss. Our hypothesis was that alignment using PMI would be at least as accurate as that obtained with standard instrumentation, without statistically important differences in operating time or blood loss.

## Patients and methods

The prospective randomized study was carried out at the Orthopaedic Department in a single university hospital (University Clinical Center Maribor, Slovenia) between June and November 2011. A total of 38 patients with advanced osteoarthritis who were waiting for primary TKA were randomly assigned to one of two groups (1:1), using either PMI—Signature^TM^ system or standard instrumentation (STD group). Exclusion criteria were a body mass index (BMI) above 40 kg/m^2^ and a preoperative mechanical axis above 20° of varus and 10° of valgus.

The Vanguard cemented knee prosthesis (Biomet, Inc., Warsaw, IN, USA) with posterior-stabilized femoral components was implanted in all patients. The medial parapatellar surgical approach without eversion of the patella and initial distal femoral cuts was used. All patients also received a single dose of cefazoline (1 or 2 g intravenously) before surgery, antithrombotic prophylaxis (dalteparin) and had no patellae resurfaced and no use of tourniquets during surgery, except in the cementation phase. All procedures were performed by one experienced surgeon.

**Fig. 1:**
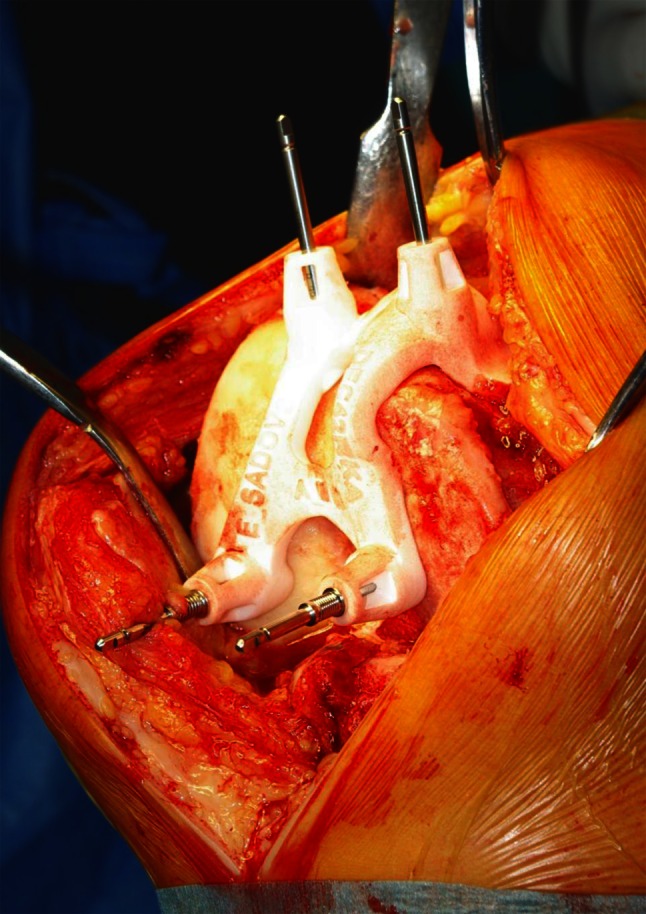
Intraoperative use of specific positioning guides for accurate pin placement

The only treatment difference between the two groups was the placement of the pins for standard cutting blocs for the femur and the tibia. In STD group, the placement of pins and cutting guides was made on a per-case basis, using standard intramedullary femoral and extramedullary tibial assembly. In contrast, in the PMI group pin placement was done with the help of specific positioning guides, a technique which allows us to determine the level and orientation of all necessary cuts to the femur and tibia. In all cases, the goal of preoperative planning was to restore neutral mechanical alignment, 3° of femoral component flexion, and neutral rotation according to the epicondylar axis. PMI patients underwent a preoperative MRI scan of hip, knee, and ankle to create virtual reconstruction of individual knee anatomy to design the 3D models of the distal femur and proximal tibia. Special software (Materialise N.V., Leuven, Belgium) was used to process the MRI scan images; thereafter, each detailed operative plan was reviewed and approved by the surgeon. With the help of rapid prototype technology, specific positioning guides were then manufactured. Intraoperatively, as much soft tissue as possible was removed in the area of intended placement of the guides on the distal femur and proximal tibia with retention of all osteophytes and remnant cartilage. Custom-made pin guides (femoral and tibial) were then placed on a “perfect fit basis” (shown in Fig. 1) to assure accurate pin placement. After placement of the pins, the standard cutting blocs were used and resection was performed in the usual manner.

Full-length, standing, anterior-posterior radiographs were taken in all patients preoperatively and postoperatively before discharge (on average 4–6th day) or after full extension was achieved. The mechanical axis was measured as the angle between a line from the center of the hip joint to the center of the tibial tray, and a line from the latter position to the midpoint of the ankle joint.

The accuracy of the surgical procedure was measured by comparing preoperative and postoperative mechanical alignment between the groups. Surgical performance was measured by blood loss and surgery time (more or less than 90 min). The former was estimated from the preoperative (on the day of surgery) and postoperative hemoglobin (Hb) levels (on the first postoperative day).

The SPSS statistical software package, version 19.0 was used for all statistical analyses. Data were expressed as the mean  ± standard deviation and median. The Wilcoxon–Mann–Whitney U test and χ^2^ test were performed to compare preoperative and postoperative data. A *P*-value less than 0.05 was considered statistically significant.

This present study was approved by the local independent ethics committee, and informed consent was obtained from all the patients included.

## Results

A total of 38 patients with advance osteoarthritis waiting for TKA—19 patients in the STD group (5 men and 14 women) and 19 patients in the PMI group (2 men and 17 women)—were included in the present study. Patients in both groups had comparable age, BMI, preoperative mechanical axis, KSS and levels of Hb (Table 1).

**Table 1:** Patients demographic data: age, body mass index (BMI), preoperative Knee Society Score (KSS), preoperative mechanical axis and preoperative hemoglobin (Hb) level

Parameters	STD group (*N* = 19)			PMI group (*N* = 19)		
	Mean ± SD					
Age	66.8 ± 6.7			67.1 ± 7.1		
BMI	33.3 ± 5.5			31.9 ± 5.3		
KSS for knee and function	49 ± 15			54 ± 18		
	63 ± 11			58 ± 21		
	Mean ± SD	95 % CI	Median	Mean ± SD	95 % CI	Median
Preoperative mechanical axis^a^	3.6 ± 2.4	2.5–4.8	3.1	4.2 ± 2.0	3.2–5.1	4.3
Preoperative Hb level	133 ± 16	125–140	137	134 ± 12	128–140	135

There were no statistical significant differences among the groups

*STD* standard TKA instrumentation, *PMI* patient-matched instruments

^a^Represented as an absolute difference from the neutral axis

**Fig. 2:**
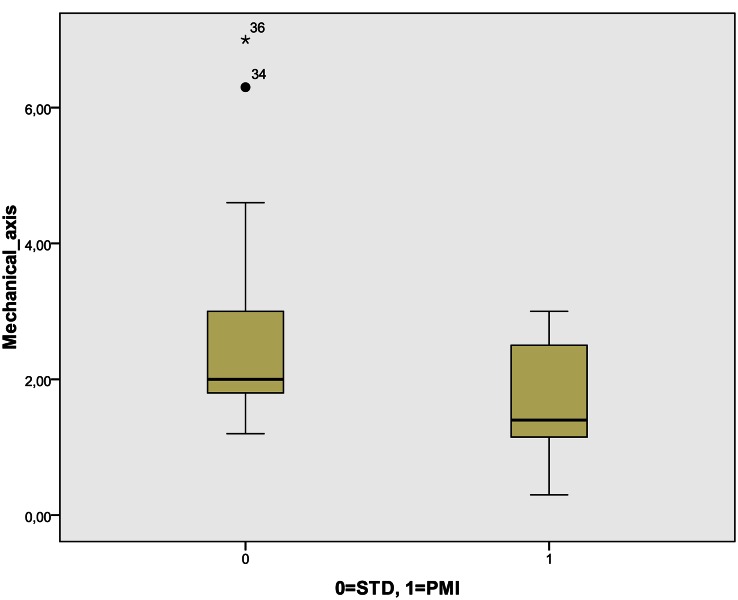
Postoperative mechanical axis between STD and PMI group. STD standard TKA surgical instrumentation, PMI patient-matched instruments

Postoperative results showed a significant difference in postoperative alignment between the groups: the PMI group was found to be closer to neutral mechanical axis as shown in Fig. 2. There were no outliers (deviation from a neutral mechanical axis for more than 3°) in PMI group and four outliers (22 %) were recorded in STD group; however, the difference was not statistically significant (*P* = 0.053). Furthermore, significant reduction in the time for the operation was observed in the PMI group. However, there was no significant difference in the measurement of blood loss (difference between preoperative and postoperative Hb). Postoperative results are presented in Table 2. There was no adverse event or complication while using patient-specific instrumentation, and all cutting blocs perfectly fitted the individual patient’s knee joint anatomy during surgery. None of the patients needed postoperative blood transfusion.

**Table 2:** Postoperative measurements among STD and PMI group: mechanical axis, difference in pre- and postoperative hemoglobin (Hb) level and operation duration

Parameters	STD group (*N* = 19)			PMI group (*N* = 19)			*P* value
	Mean ± SD	95 % CI	Median	Mean ± SD	95 % CI	Median	
Mechanical axis	2.7 ± 1.7	1.9–3.5	2.0	1.7 ± 0.9	1.3–2.1	1.4	0.013^a^
Difference in Hb	31.0 ± 3	25–37	28	28 ± 2	24–32	29	NS
Operation duration	< 90 min		9 (47 %)	< 90 min		16 (84 %)	0.017^b^
	> 90 min		10 (53 %)	> 90 min		3 (16 %)	
Number of outliers	Deviation less than 3°		15 (79 %)	Deviation less than 3°		19 (100 %)	0.053^c^
	Deviation more than 3°		4 (21 %)	Deviation more than 3°		0 (0 %)	

*STD* standard TKA instrumentation, *PMI* patient-matched instruments, *NS* = not significant

Statistical tests: ^a^Mann–Whitney U test (one-tailed significance), ^b^χ^2^ test, ^c^Fisher’s exact test (one-sided)

## Discussion

PMI are disposable custom guides made on an individual basis for accurate pin placement to assure exact positioning of standard resection instruments and to prevent malalignment of the prosthesis. However, there is still a limited amount of published peer-reviewed clinical data supporting the advantages of PMI. The current study, therefore, addressed the surgical accuracy and performance of the novel technology of specific patient-matched pin guides.

The important finding of the present study was the advantage of PMI in determining neutral mechanical axis of the prosthesis; PMI patients showed a mechanical axis significantly closer to neutral (1.7 versus 2.7°, *P* = 0.013) and no outliers, compared with the STD group. The results of the present study are consistent with recent reports [26–28], which demonstrated the superior value of PMI in early postoperative alignment and in preventing malalignment. However, recent preliminary experience of the PMI system showed no benefit in alignment accuracy based on data acquisition with A-P radiograms [29].

Despite the uncertainty about the true effect of alignment on patient outcome [30–32], malalignment exceeding 3° of varus/valgus in regard to mechanical neutral is still associated with increased risk of failure [11–14, 32] and also with inferior functional outcomes [22]. Current techniques with standard TKA instruments violate intramedullary canals; additionally, with this method accuracy in aligning the mechanical axis within 3° varied in the literature [17–21] and was inferior compared with the results of computer-assisted TKAs [23–25]. Nevertheless, the latter has limitations in terms of increased surgical time (pin placement, landmark registration), costs, potential complications (pin loosening, pin-related bone fracture, exposure-related infection) and a substantial learning curve [26]. Intramedullary violation with the standard technique could also lead to higher blood loss and an increased risk of fat embolisms [33]. However, further long-term studies should be conducted to confirm the long-term effectiveness of PMI and to verify whether aligning the knee closer to neutral promises better long-term outcomes. Several studies have reported that as well as neutral mechanical axis in the frontal plane, accurate alignment in the sagittal and horizontal planes does play an important role in long-term outcomes [34–37]. Regarding the debate on alignment’s influence on the long-term outcome [30–32], it would be interesting to assess 3D alignment of the prosthesis and to evaluate the short- and long-term efficiency of PMI in restoring good position and stability of the endoprothesis.

Another important finding in the present study involved the significant reduction in the time of surgery in the PMI group compared with the STD group. A similar result was also demonstrated by other researchers; in addition, reductions in the number of instrument trays, duration of hospital stay and incision length were also reported, with a beneficial impact on cost reduction [26, 27, 38]. However, this was not investigated in the present study. Several other advantages of PMI were discussed: e.g., the decreased duration of surgery might decrease the risk of infection and blood loss; no reaming in the intramedullary canals might minimize the risk of postoperative complications such as fat embolism. Certainly, the results of the present study showed no difference in blood loss between the PMI and STD groups.

In conclusion, PMI can contribute in achieving better mechanical axis with reduction in outliers and decreased operation time, as it was also shown in the present study. However, differences are small and therefore it would be interesting to design a randomized long-term study with a large cohort of participants to evaluate whether PMI can ensure better survival of the prosthesis after TKA, improves clinical function and patient satisfaction, in addition to preventing perioperative and postoperative complications, and whether its routine use is justified in primary TKA. Despite constant improvements in TKAs regarding design and type of implant, materials and implant instruments, we believe that excellent surgical experiences still remains the crucial factor in the long-term efficiency after TKA. Nevertheless, it is still essential to continue improving and reliably reproducing the overall survivorship of this procedure, and the use of new technologies such as PMI could contribute to this goal.
